# Research Progress on the Roles of Cytokinin in Plant Response to Stress

**DOI:** 10.3390/ijms21186574

**Published:** 2020-09-08

**Authors:** Yun Liu, Mingjing Zhang, Zhe Meng, Baoshan Wang, Min Chen

**Affiliations:** Shandong Provincial Key Laboratory of Plant Stress Research, College of Life Science, Shandong Normal University, Jinan 250014, China; m17853729019@163.com (Y.L.); m15165085813@163.com (M.Z.); zmeng@sdnu.edu.cn (Z.M.)

**Keywords:** abiotic stress, cytokinins, plant resistance, research progress

## Abstract

Cytokinins promote plant growth and development under normal plant growth conditions and also play an important role in plant resistance to stress. Understanding the working mechanisms of cytokinins under adverse conditions will help to make full use of cytokinins in agriculture to increase production and efficiency of land use. In this article, we review the progress that has been made in cytokinin research in plant response to stress and propose its future application prospects.

## 1. Introduction

Cytokinins are one of the five major types of plant hormones that play an important role in the cell cycle and affect the growth and development of plants [1]. In addition to promoting cell division and plant growth and development, cytokinins also impede plant senescence by inhibiting the decomposition of chlorophyll, nucleic acids, proteins, and other substances in plants and redistributing necessary amino acids, hormones, inorganic salts, and other compounds to other parts of the plant [2]. An increasing amount of research shows that cytokinins can alleviate the damage to plants caused by a variety of abiotic stresses [3,4,5]. However, under adversity stress, mechanisms of cytokinin-alleviating stress are different under different stresses. Can the mechanism of cytokinin alleviating one stress be used for other stresses and can it be understood from the perspective of cross adaptation? In this review, cytokinin synthesis, metabolism, signaling pathways, and research progress on these topics in regards to plant resistance to stress (cold, heat, salt, and drought stress) and its future application prospects are discussed.

## 2. Synthesis and Metabolism

Almost all organisms produce cytokinins (CKs). Natural cytokinins are adenine derivatives with isoprenoid or aromatic side chains at the N_6_ position of the adenine ring [6]. Therefore, natural cytokinins can be divided into isoprenoid and aromatic CKs, where the former is more commonly found in plants and more abundant than the latter [7]. Isoprenoid CKs are primarily composed of iP (isopentenyl adenine), tZ (trans-zeatin), cZ (cis-zeatin), and DZ (dihydrozeatin), of which iP and tZ are considered the main active cytokinins [7,8]. The aromatic cytokinins include orthotopolin (oT), mesotopolin (mT), their methoxy derivatives (MeoT and MemT, respectively), benzyladenine (BA), and others. These, however, are only found in some plant species such as poplar and tobacco [7,8]. In addition to natural cytokinins, there are also some synthetic cytokinins, such as kinetin, 6-benzylaminopurine (6-BA), benzyladenine, and trans-zeatin riboside that exhibit cytokinin activity and can affect plant growth via an external application.

**Figure 1:**
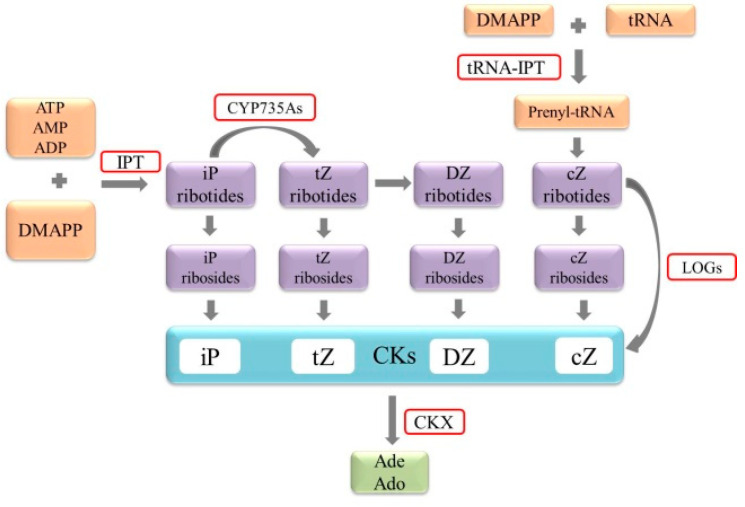
Simplified model of cytokinin biosynthesis and decomposition pathways.

Synthetic precursors of cytokinin and its synthesis process rely on the terpenoid pathway. The key enzymes involved in cytokinin biosynthesis are isopentenyl transferase (IPT) and LONELY GUY (LOG) (Figure 1) [9]. IPTs are the central rate-limiting enzymes for cytokinin biosynthesis and are composed of adenosine phosphate-isopentenyl transferases and tRNA-isopentenyl transferases (tRNA-IPTs). The common feature of these two types of IPTs is a conserved IPPT-binding domain [7]. The iP-ribotides are precursors of iP [8]. The adenosine phosphate-isopentenyl transferases can catalyze dimethylallyl diphosphate (DMAPP) to form iP-ribotides, which initiates cytokinin biosynthesis [7]. Then iP-ribotides are catalyzed by cytochrome P450 monooxygenase CYP735As into tZ-ribotides [10] and this process also plays an important role in promoting the growth of *Arabidopsis* shoots [6]. These iP- and tZ-ribotides are the precursors of most iP-type and tZ-type cytokinins, which can generate iP and tZ through ribosides [8]. The biosynthesis of cZ is initiated by tRNA-IPTs, which use DMAPP to catalyze the prenylation of tRNA and then further generate cZ-ribotides (Figure 1) [7]. IPTs are encoded by multiple gene families in some plants [11], for example, 9 *IPTs* (*AtIPT1-AtIPT9*) have been identified in *Arabidopsis* [11], 10 *IPTs* (*OsIPT1*-*OsIPT10*) in rice [12], and 7 *FvIPTs* in the strawberry genome [3]. LOGs are a new cytokinin-activating enzyme with a conserved lysine decarboxylase-binding domain in all the proteins expressed by LOGs [3]. Through its cytokinin-specific phosphohydrolase activity, LOGs directly convert the inactive cytokinin ribotides into a biologically active free base form [8]. *LOGs* were first discovered in rice, and the nine *LOGs* (*AtLOG1-AtLOG9*) found in *Arabidopsis* were later considered to be the rice homologs [9]. The strawberry genome recently identified nine *FvLOGs*. The expression of most *FvIPTs* and *FvLOGs* changes under osmotic stress, high temperature treatment, and exogenous abscisic acid (ABA), suggesting that these genes may play a role in plant resistance to abiotic stresses [3].

The maintenance of cytokinin in plants requires fine control of the biosynthesis and metabolic enzymes. Therefore, the maintenance of stable cytokinin content requires not only cytokinin synthetase but also cytokinin-metabolizing enzymes [13]. The level of active cytokinin can be regulated by binding to sugars (most commonly glucose) or by irreversible cleavage of cytokinin oxidases (CKXs) [14]. The binding of glucose to cytokinin occurs at the N^3^, N^7^, and N^9^ positions of the purine ring or the hydroxyl group of the pentenyl side chain, including O and N-glycosylation [8]. O-glycosylation occurs on the oxygen of the cytokinin side chain, which is catalyzed by glucosyltransferase and reversed by β-glucosidase [7]. N-glycosylation, which mainly occurs on the N^7^ or N^9^ of the purine ring, is considered irreversible [7,8]. Glucosyl conjugates are not active in bioassays and these bound cytokinins cannot bind to histidine kinase (HK) cytokinin receptors [15]. CKXs are the cause of irreversible degradation of cytokinin and the only known enzyme that specifically catalyzes this process [8,16]. Mature CKXs contain two conserved domains, FAD (flavin adenine dinucleotide) binding and substrate binding, which have different subcellular localization and biochemical functions [16,17]. CKXs reduce the level of active cytokinins by irreversibly cutting the free radicals and ribose forms of cytokinins on the N^6^ side chain [14]. Both tZ and iP are cleaved by CKXs, but DZ and synthetic cytokinin kinetin and 6-benzylaminopurine are resistant to CKXs cleavage [16]. It is worth noting that the overexpression of *CKXs* results in a decrease of endogenous cytokinin levels and various growth and developmental defects [18]. *CKXs* have been detected in many species, including five *CKXs* members (*ZmCKX1*-*ZmCKX5*) in maize and seven *CKXs* (*AtCKX1*-*AtCKX7*) in *Arabidopsis* [19].

The orange solid box represents the synthetic precursor of cytokinin; the purple solid box represents the intermediate metabolite during the synthesis process; the blue solid box represents cytokinin; the green solid box represents the metabolite of cytokinin. The red boxes indicate some key enzymes in synthesis and metabolism. CKX, cytokinin oxidase/dehydrogenase; cZ, cis-zeatin; DMAPP, dimethylallyl diphosphate; iP, isopentenyl adenine; DZ, dihydrozeatin; tZ, trans-zeatin; IPT, adenosine phosphate-isopentenyl transferase; LOG, LONELY GUY; tRNA-IPT, tRNA-isopentenyltransferase; Ade, adenine; Ado, adenosine.

## 3. Cytokinin Signaling Pathway

**Figure 2:**
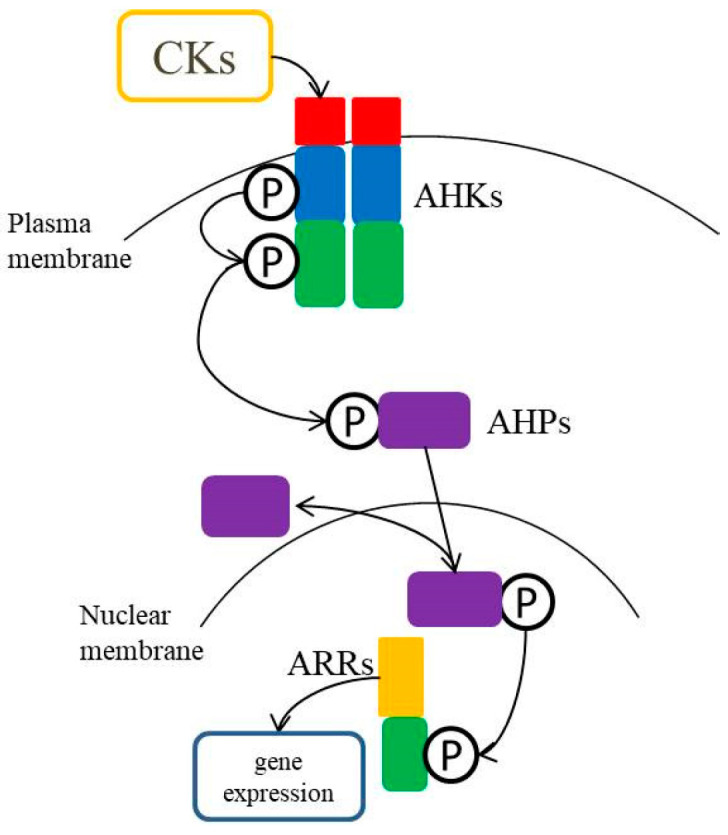
Diagram of the composition of the plant multi-step phosphorylation system.

To date, the cytokinin signal transduction pathway has been thoroughly studied. Microorganisms use a two-component system (TCS) to change gene expression levels to respond to various stimuli and improve their ability to perceive and adapt to environmental changes [20,21]. This two-component system includes the following two proteins: histidine kinases (HKs) associated with the membrane and response regulators (RRs) in the cytoplasm. HKs detect the environmental input in the sensor area and transmit the generated signal to the cytoplasm [21]. Based on TCS, plants have evolved a multi-step phosphorylation system, including the following three components: HKs, histidine phosphotransfer proteins (HPs), and RRs (Figure 2) [22]. This multi-step phosphorylation system usually involves continuous phosphorylation events that alternate between histidine (His) and aspartate (Asp) residues [23]. Cytokinin uses this multi-step phosphorylation system for its signal transduction, including participation in cell division, leaf senescence, and apical dominance [24]. In addition, this multi-step phosphorylation system is also an important way for plants to transmit stress signals via cytokinins [25].

Cytokinin signal transduction has been studied in detail in *Arabidopsis thaliana*. A variety of HKs have been found as cytokinin receptors, including *Arabidopsis* histidine kinase 2 (AHK2), *Arabidopsis* histidine kinase 3 (AHK3), and cytokinin response 1 (CRE1)/wooden leg1 (WOL1)/*Arabidopsis* histidine kinase 4 (AHK4), which are primarily located in the plasma membrane [23,26]. The overall structure of HKs includes a kinase domain-containing His residue, a receptor (Rec) domain, an N-terminal extracellular signal input domain, and several transmembrane domains [27,28]. Cytokinin acts as a signal to the HKs signal input domain, which results in the autophosphorylation of the conserved His residue in the kinase domain. The phosphate group is then transferred to the conserved Asp in the Rec domain [26] and again transferred to the downstream HPs and RRs to form a regulatory circuit. The cytokinin signal eventually leads to changes in transcription in the nucleus [29]. It has recently been shown that the cyclase/histidine kinase associated sensory extracellular (CHASE) domain-containing HKs are not limited to terrestrial plants, but may also appear in other eukaryotes (fungi and amoeba) and prokaryotes (plant pathogens and cyanobacteria) [30]. For example, the plant pathogen *Xanthomonas campestris* recognizes plant-derived cytokinins through a CHASE domain, suggesting that the cytokinin signaling system is not only adapted by plants, but also by different species to regulate cell processes, and plays a role in plant–microbe interactions and stress adaptation [31].

Cytokinin binds to the receptor (AHKs), AHKs autophosphorylates, and then the phosphate group is transferred to downstream AHPs and ARRs to form a regulatory circuit, leading to the related gene expression. AHKs, *Arabidopsis* histidine kinases, including AHK2, AHK3, AHK4/CRE1/WOL1; AHPs, *Arabidopsis* histidine phosphotransfer proteins, including AHP1-AHP5 and APHP1/AHP6; ARRs, Arabidopsis response regulators, including A-type ARRs (ARR3-9, 15-17), B-type ARRs (ARR1, 2, 10-14, 18-21), C-type ARRs (ARR22 and ARR24).

Similar to bacterial and yeast HPs, plant HPs play a role in the multi-step phosphorylation signaling pathway [32]. AHPs (*Arabidopsis* histidine phosphotransfer proteins) are a family of six related proteins that contain a highly conserved motif (XHQXKGSSXS), which is necessary to transfer the phosphate group from the Rec domain of HKs to the Rec domain of RRs [27]. AHP1-AHP5 contains the conservative amino acids required for the function of AHPs and provides a mobile connection between the cytoplasm and nucleus and continuous circulation. Sensitivity of different mutants of five typical AHPs to cytokinin was decreased, which suggested that most of the AHPs were positive regulators of the cytokinin signal and there is a functional overlap between the AHPs [32]. However, APHP1/AHP6 (a pseudo-AHP) lacks a conserved histidine residue that is a phosphorylation target and cannot be used as a phosphotransfer protein [33,34]. HPs have no catalytic activity and are fundamentally a high-energy phosphorylation donor of RRs. HPs act downstream of HKs in cytokinin signal transduction. After being phosphorylated by HKs, HPs can phosphorylate RRs, mediating the transfer of phosphate from the Rec domain of activated HKs to the Rec domain of RRs [32]. HPs have a conserved cysteine residue. If the cysteine residues are nitrogenated by nitric oxide, the ability of HPs to act as a phosphate transfer protein is inhibited [35].

RRs, as the terminal components of TCS, mediate the expression of downstream genes and play an important role in abiotic plant stress response. RRs have been extensively studied in *Arabidopsis* and according to conserved sequences, characteristics, and domains, ARRs can be divided into four types: A-type ARRs, B-type ARRs, C-type ARRs, and *Arabidopsis* pseudo-RRs (APRRs). B-type ARRs (ARR1, 2, 10-14, 18-21) include an N-terminal Rec domain and a large C-terminal region, and the C-terminal region contains a Myb-like DNA binding domain and a transcription activation domain [36]. In the nucleus, AHPs transfer phosphate groups to Asp residues in the Rec domain of B-type ARRs to activate B-type ARRs, essential for the initial transcription reaction of cytokinins [37,38,39]. B-type ARRs are positive regulators of cytokinin signal response. Studies have shown that the triple mutant *arr1 arr10 arr12* severely weakens the expression of most cytokinin regulatory genes and makes plants insensitive to cytokinin [38]. Therefore, it is believed that cytokinin regulates the downstream response through a multi-step phosphorylation system, with ARR1, ARR10, and ARR12 playing a central role in regulating the transcription and physiological response of cytokinin. A-type ARRs (ARR3-9, 15-17) consist of a Rec domain, short N-terminal, and short C-terminal extensions [40]. According to reports, A-type ARRs play a negative regulatory role in the cytokinin signaling pathway [41]. B-type ARRs are direct upstream activators of cytokinin-induced A-type ARRs [42,43]. However, the mechanism of negative regulation of cytokinin signal transduction by A-type ARRs is unclear, which may involve phosphorylation competition of B-type ARRs [44]. The domains of C-type ARRs (ARR22 and ARR24) are similar to A-type ARRs, however, the expression of C-type ARRs is not cytokinin-induced [45] and their role in cytokinin signaling is unclear [24]. APRRs lack highly conserved Asp residues required for phosphorylation [46] and the C-terminus of APRRs contains a specific CCT (Co, Col, and Toc1) motif [46,47].

## 4. Role of Cytokinin in Abiotic Stress Response

Cytokinins are involved in many aspects of plant growth and development. Cytokinin research is progressing rapidly with a large number of mutants produced in the cytokinin signaling pathway, synthesis, and decomposition process. Studies show that cytokinins play an important role in the plant response to abiotic stress.

### 4.1. Heat Stress Response

Heat stress reduces chlorophyll content and photochemical efficiency (Fv/Fm) of leaves, which has a negative impact on the photosynthetic capacity [48]. Heat stress also increases the production of ROS (reactive oxygen species) and protease activity, leading to leaf senescence [49]. Under heat stress, endogenous cytokinin in *Arabidopsis* increases, particularly in the leaves [50], leading to the hypothesis that increased cytokinin content is responsible for the increase in heat tolerance [51]. Heat stress increases the production of ROS and elevated cytokinins can stimulate the antioxidant system to remove ROS [52]. The analysis of hormones, proteome, and transcriptome also confirms that cytokinin plays an important role in plant resistance to heat stress, and most of the heat shock (HS) response proteins are upregulated by increased cytokinin [51]. However, when the cytokinin signaling pathway is damaged and/or the level of endogenous cytokinin is reduced, the elongation of hypocotyls in *Arabidopsis* seedlings is significantly and continuously inhibited under heat stress, as well as during the subsequent seedling growth [53]. The accumulation of endogenous cytokinins can maintain normal plant growth under high temperature stress and have a positive impact on plants treated with heat shock [51]. Therefore, heat stress tolerance of plants can be improved by increasing the content of endogenous cytokinin.

Insertion of *isopentenyl transferase* (*IPT*) in *Arabidopsis* seedlings significantly improves the level of endogenous cytokinin and thus enhances the tolerance to high temperatures [51]. Skalak et al. [51] found that the duration of high levels of cytokinin is critical to heat tolerance in plants. The maintenance of high levels of cytokinin can be obtained through the overexpression *IPT* gene under the initiation of continuous induction of expression promoters, such as senescence-activated promoter (*SAG12*) or *HSP18* promoter. Xu et al. [52] found that the overexpression of *SAG12:ipt* in creeping bentgrass (*Agrostis stolonifera*) maintains the formation and elongation of roots under heat stress, reduces the loss of chlorophyll, and delays the senescence of leaves, enhancing plant heat resistance [52]. Later, Xu et al. found that the overexpression of *IPT* induced by two different promoters (*SAG12:ipt*, *HSP18:ipt*) leads to a significant increase in heat stress proteins in plants, which in turn increases their heat tolerance [54]. The overexpression of the *IPT* gene in *Arabidopsis* driven by dexamethasone (DEX) promoter (a kind of transient expression promoter) can result in stomatal opening and stimulation of leaf transpiration, which are crucial in the early stage of HS response [51]. While the improvement in transpiration removes a portion of the heat away to maintain the normal temperature of plants, it also promotes the transport of cytokinins from roots to shoots through the xylem [55]. However, due to the limited water content of plants, enhancement of transpiration can only alleviate the urgent need for cooling and cannot improve the long-term heat stress effects.

In addition to increasing the endogenous cytokinin content through overexpression of *IPT*, maintaining a high level of cytokinin can be achieved by reducing the degradation of endogenous cytokinin. There are two ways of inhibiting the degradation of endogenous cytokinin. One is by the CK oxidase/dehydrogenase (*CKX*) gene mutation, while the other is to spray cytokinin degradation inhibitors to inhibit CK oxidase/dehydrogenase (a negative regulator of cytokinin synthesis) activity. Mutation of the *CKX* gene in rice results in the increase of cytokinin level and grain yield. In addition, the CKX activity of heat-sensitive rice varieties increases significantly, resulting in low cytokinin levels and rice yield. The CKX enzyme activity of heat-resistant rice keeps stable and heat-resistant rice increases heat resistance [55]. INCYDE (cytokinin degradation inhibitor) is an effective *CKX* inhibitor. Under heat stress, the application of INCYDE can increase the content of active cytokinin in *Arabidopsis* roots [56]. However, Prerostova et al. [57] observed the opposite result and a single INCYDE treatment under heat stress negatively impacts heat tolerance in plants. The combination of utilizing INCYDE with acclimating plants can partially promote *Arabidopsis* heat tolerance. Cytokinin response factors (CRFs) are considered to be a cytokinin related transcription factor. The expression of *CRF1* in tomato roots significantly downregulated under heat stress [58]. Based on this, we believe that CRFs play an important role in the plant’s heat stress signal pathway and further research is needed to clarify the underlying mechanism.

Under heat stress, exogenous cytokinin has a similar function to endogenous cytokinin. The application of exogenous cytokinin zeatin ribose (ZR) in creeping grass increases the heat tolerance, manifesting in the reduction of root mortality, the maintenance of higher chlorophyll content [49], increased the activity of antioxidant system [52], and the upregulation of corresponding heat shock proteins [59]. In addition, exogenous cytokinin can also enhance the tolerance of plant reproductive organs under heat stress to improve plant yield. In the fruits of rice, *Arabidopsis*, and passion fruit (*Passiflora edulis*), exogenous cytokinins can alleviate the negative effects of heat stress on branches and florets and increase their yield [55,60,61].

### 4.2. Cold Stress Response

Low temperatures affect cells by hardening the membrane system and interfering with all membrane-related processes [62]. Low temperatures can also lead to the accumulation of ROS, due to the decrease of antioxidant enzyme activity making the ROS scavenging system unable to work normally, and in turn, the excessive accumulation of ROS will have harmful effects on the membrane, resulting in ion leakage and cell metabolism disorder [63]. Low temperatures also inhibit reproductive development. For example, exposure of the flowering stage of rice to cold stress will cause sterility and result in yield loss [64,65]. In addition, freezing stress occurs when the temperature is lower than 0 °C and the developing ice crystals will cause mechanical damage and metabolic dysfunction of plants [66,67,68].

Cold stress exhibits a significant strain on energy production and biochemical demand [69]. Therefore, compared with low latitudes (relatively high-temperature area), Zoysia grass (*Zoysia japonica*) at high latitudes (relatively low-temperature area) displays higher frost resistance. This is possibly due to the higher carbohydrate content used as an energy reserve and the role of plant hormones in regulating plant adaptation to low temperatures [70]. Under low-temperature stress, cytokinin content in *Carpobrotus edulis* increases [71] and an *Arabidopsis* mutant *amp1* with higher cytokinin levels can display a better relative growth rate and higher plant yield than wild-type [72,73]. At the same time, overexpression of *AtCOR15a*:*ipt* in sugarcane improves tolerance to low temperatures by slowing leaf senescence and reducing membrane damage, avoiding severe productivity loss and freezing injury [74].

Based on the role of cytokinins in low-temperature stress, many researchers have used cytokinin to improve plant tolerance to low-temperature stress. At present, the research on the relationship between low-temperature resistance and cytokinins has primarily focused on the multi-step phosphorylation system. Some studies have shown that the A-type *ARRs*—such as *ARR5*, *ARR7*, and *ARR15*—are positive regulators of *Arabidopsis* cold tolerance [75]. *ARR22*, a C-type *ARR*, also plays an active role in plant low-temperature resistance by maintaining the normal state of the membrane [76]. Compared with the wild-type, the B-type *arr1* is sensitive to low temperature with reduced cold resistance, while the *Arabidopsis* with B-type *ARR1* overexpression shows increased cold resistance, suggesting that *ARR1* is a positive effector of cold signal transmission [77]. ARR1 receives low-temperature signals from AHK2 and AHK3 mediated by AHP2, AHP3 or AHP5, showing that AHP2, AHP3, and AHP5 also play an active role in upregulating resistance to low-temperature stress [77]. Other cytokinin response factors (CRFs), which may be located downstream of the cytokinin signaling pathway, also affect the low-temperature resistance of plants. *Arabidopsis* with *CRF4* mutation under low-temperature stress shows that *CRF4* is a positive regulator for the enhancement of cold tolerance [78]. The high expression of *CRF2* and *CRF3* under cold stress may be an adaptive mechanism under cold stress, which can promote the initiation and development of lateral roots, to overcome the inhibition of cold-induced root growth, and enhance the cold tolerance of plants [79]. However, it has been reported that cytokinin receptor histidine kinases (*AHK2, AHK3, AHK4*) and *ARR7* (an A-type *ARR*) play a negative regulatory role in cold stress signaling, and the mutations of *AHK2, AHK3, AHK4,* and *ARR7* lead to increased low-temperature resistance in *Arabidopsis* [80]. However, further experimental verification is needed.

The increase of cytokinin, whether exogenous or endogenous, can improve low-temperature resistance [74,77]. Pretreatment with exogenous cytokinin can improve the cold tolerance of wheat seedlings under cold stress by increasing the endogenous cytokinin in leaves [81]. The application of exogenous cytokinin in *ahk* mutant is similar to the higher-order mutation of AHK (a negative regulatory factor) to some extent under cold stress, which can improve plant cold tolerance. However, the molecular mechanisms are still unclear [80]. It has been suggested that some A-type *ARRs* are positive regulatory genes of cold stress and that exogenous cytokinin treatment enhances the cold tolerance of wild-type plants. Type-A *ARRs* are highly expressed in transgenic plants via stabilizing ARR protein [75].

Ethylene and cytokinin signals are antagonistic to cold stress. The best known cold signaling pathway is the C-repeat binding factor/DRE binding factor (CBF/DREB) transcription regulatory cascade. Overexpression of CBF can lead to sustained increased cold tolerance [82]. Ethylene negatively regulates the cold signal by regulating the expression of CBFs and A-type *ARRs* genes, and A-type *ARRs* are considered to be the key to integrating ethylene and cytokinin signals in regulating plant response to cold stress [75].

### 4.3. Salt Stress Response

Under salt stress, various physiological and biochemical processes in plants are being affected. Sodium-ion (Na^+^) accumulation in plants can lead to the disorder of ion homeostasis, the imbalance of potassium ion (K^+^)/Na^+^ ratio, and Na^+^ ion toxicity [83,84,85,86,87], which cause secondary stress including oxidative stress. Oxidative stress causes cell membrane damage, ion leakage, or direct damage to proteins and other macromolecules leading to cytotoxicity, membrane dysfunction, and even cell death [88,89,90,91]. Ion stress and oxidative stress will accelerate leaf senescence by degrading chlorophyll, inhibiting photosynthesis, and reducing yield [87,92,93,94,95]. Some studies have shown that the negative effects of salt on plants such as radish and tobacco are related to cytokinins [96]. However, the changes in endogenous cytokinins in different plants are not uniform under salt stress. Due to the variations of cytokinin content under salt stress, there is no unique way to improve plant salt tolerance by changing endogenous and exogenous cytokinins.

Some studies have shown that under salt stress, the cytokinin content of apple rootstock ‘robusta’ remains high [97] and the cytokinin level in tomato seedlings also increases [4]. In addition, the cytokinin levels related to salt stress also increase in rice, *Arabidopsis*, and other plants [98,99]. In some plants, the upregulation of cytokinin can alleviate damage from salt stress. *OsCKX2* knockout rice mutant, with high cytokinin level, has higher relative water content and yield under salt stress compared to wild-type via cytokinin accumulation and thus improving salt tolerance [99]. Spraying INCYDE on tomato under salt stress can improve plant salt tolerance by increasing the activity of antioxidant enzymes [100]. Exogenous methyl jasmonate (MeJA) pretreatment of wheat can maintain the high content of cytokinin by decreasing *CKX* transcription level induced by salt stress, reduce the delay of salt on seedling growth, and enhance salt tolerance [101]. Due to the deletion of 42 bp in the promoter region of the *IPT5* gene, the expression level is increased along with the cytokinin content of apple rootstock ‘robusta’ under salt stress which was maintained at a high level and enhanced salt tolerance [97]. High cytokinin content in the tomato of *SlIPT3* overexpression significantly improves salt tolerance by maintaining normal photosynthetic pigment and a high K^+^/Na^+^ ratio [102].

On the other hand, studies have shown that high cytokinin levels decrease plant salt tolerance. Overexpression of *AtIPT8* in *Arabidopsis*, with high cytokinin content, causes a significant decrease in the survival rate of plants under salt stress by downregulating the expression of stress-responsive genes, inhibiting the antioxidant system, and reducing chlorophyll content [103]. In addition, it has been found that plants with reduced cytokinin levels have increased tolerance to abiotic stresses, including salt stress, due to the reduced cytokinin synthesis or increased degradation [101,102,104,105]. Mutants with a loss-of-function mutation in genes involved in the cytokinin synthesis pathway—such as *Atipt1*, *Atipt3*, *Atipt5*, and *Atipt7*—exhibit a stronger salt-tolerant phenotype than the wild-type [104,106]. Overexpression of *PpCKX1* in the moss *Physcomitrella patens* causes cytokinin levels to decrease and exhibit higher salt tolerance [107]. *MsCKX* overexpression enhances the salt tolerance of transgenic alfalfa plants by maintaining a high K^+^/Na^+^ ratio and enhancing the activity of antioxidant enzymes to scavenge ROS [108]. Compared with *ipt* mutants, these *CKX*-induced cytokinin-deficient plants are more useful for studying the role of cytokinin [109].

The elements in the cytokinin signaling pathway play important roles in plant salt tolerance. Cytokinin receptor AHK1 plays an active regulatory role in osmotic stress signaling and acts as a positive regulator of the salt stress response [110]. However, *ahk2*, *ahk3*, and *cre1* mutants enhance plant salt tolerance by upregulating the expression of corresponding stress response genes, showing that these members play a negative regulatory role in salt tolerance [110]. The *arr1* and *arr12* mutants decrease the accumulation of sodium in the aerial parts and enhance the salt stress tolerance by promoting the expression of *Arabidopsis high-affinity K^+^ transporter 1;1* (*AtHKT1;1*) in the roots [111]. In addition to cytokinin content, the distribution of cytokinin may be an important factor affecting plant salt tolerance. Yin et al. [112] showed that overexpression of *ARGONAUTE2* (*AGO2*) in rice can decrease cytokinin content in shoots and increase cytokinin content in the roots, leading to increased salt tolerance and grain length of rice under salt stress. AGO2 affects the distribution of cytokinin by promoting the expression of *BIG3* (*GRAIN3*), which encodes a protein that may be involved in cytokinin transport, and AGO2, which changes the histone methylation level of BG3. Some members of CRFs are considered to be downstream signaling molecules of RRs [113]. Compared to wild type, *Atcrf1* and *Atcrf 2* mutants had high photosystem II efficiency and yield under salt stress [114]. However, it was found that RNAi silencing of *ThCRF1* under salt stress decreased the salt tolerance of *Tamarix chinensis* (a kind of halophyte), while overexpression of *ThCRF1* significantly enhanced the salt tolerance of *Tamarix chinensis* by regulating osmotic potential and enhancing the activity of antioxidant enzymes [115].

Salt tolerance is enhanced either with up or downregulation of cytokinin, depending on the plant species, the degree of salt stress, and the duration of salt stress. Treatment of plants with exogenous cytokinin also has a variety of effects on the salt tolerance of different species. Pretreatment of legumes with exogenous cytokinins increases their sensitivity to salt [116], however most studies showed that application of exogenous cytokinins enhances the salt resistance of plants, especially in some cereal crops such as wheat and rice [117,118]. Application of 6-BA can improve the salt tolerance of eggplant and perennial ryegrass by effectively alleviating salt-induced leaf senescence and other types of developmental or physiological damage [119,120]. Therefore, we can spray exogenous cytokinins to plants to increase their salt tolerance.

### 4.4. Drought Stress Response

Drought can have a variety of adverse effects on plant physiological functions, including a reduction in photosynthesis, crop yield decline, accelerated senescence, and others [121,122,123]. Similar to salt stress, the possibility of improving plant drought tolerance by regulating cytokinin levels depends on stress duration, soil water potential, and plant dehydration rate [124]. In response to drought, both up and downregulation of endogenous cytokinin have been reported to enhance drought tolerance [104,125].

Some studies have shown that during drought stress, the accumulation of plant endogenous cytokinins is reduced [126], and this reduction can enhance the plant drought tolerance via various physiological responses including stomatal closure [127], promotion of early leaf senescence, and leaf abscission [128,129,130]. Since cytokinin is a negative regulator of plant root growth and branching, it can produce plants with an enlarged root system by promoting the degradation of cytokinin in the root, improve the root to shoot ratio, and display long-term drought resistance [131,132]. Downregulation of cytokinin can be achieved by overexpression of *CKX* [125], which leads to slower plant growth and increased protective compound content (betaine, proline, etc.), and drought tolerance of *Arabidopsis* [125,133], chickpea [134], tobacco [135], and barley [131,132]. The *ipt1*, *3*, *5*, and *7* mutant genotypes in *Arabidopsis* also display a reduced endogenous cytokinin content and enhanced drought tolerance [133]. Cytokinin is downregulated, leading to the expansion of the root system and a high root to shoot ratio, which increases the water absorption area of roots. Relatively small shoots and leaf area compared to roots can effectively decreases transpiration [5,125,136]. Therefore, the whole plant can maintain relatively high relative water content and improve drought tolerance. In addition, the downregulation of cytokinin may also lead to an increase in drought tolerance by countering the effects of the oxidase system [136]. In plants, cytokinin signaling is mediated by a typical multi-step phosphorylation system that includes HKs, HPs, and RRs. Because *ahk2* and *3*; *ahp2*, *3*, and *5*; *arr1*, *10*, and *12*; and other mutants show a strong drought-tolerance phenotype, the cytokinin signaling component is also considered to be a negative regulator of drought resistance [137,138]. Therefore, it is thought that the decrease of cytokinin content can improve plant survival rate by weakening the inhibitory effect of cytokinin signal on the expression of stress response genes [104]. In addition, under drought stress, it was found that the expression of *SlCRF1*, *SlCRF2*, *SlCRF3*, and *SlCRF5* was regulated during the drought and recovery period in tomato plants, which showed that CRFs response to drought stress and provided a new idea for enhancing the tolerance of plants to drought stress [58,139].

Other studies have noted that the increase of endogenous cytokinin content can also improve the tolerance to drought stress [140]. The increase of cytokinin content may contribute to drought tolerance by inhibiting drought-induced leaf senescence. Some studies have shown overexpression of *IPT* increases cytokinin and improves drought tolerance via enhanced antioxidant system activity, photosynthesis, and the accumulation of metabolites in creeping bentgrass and *Agrostis stolonifera* [141,142], tobacco [128,143], peanut [144], and cotton [145]. The inoculation of some cytokinin-producing microorganisms can increase cytokinin content and enhance drought tolerance. For example, the inoculation of osmotolerant cytokinin producing microbes can increase the yield of tomato by improving the photosynthetic index and relative water content of plants under drought stress [146]. Inoculation of *Methylobacterium oryzae* into lentil results in significant improvement in the growth performance of plants exposed to drought, including early seedling growth, and improvement of the harvest index [147]. Under drought stress, leaf application of cytokinin and rhizobacteria (*Azospirillum brasilense* and *Rhizobium pisi*) can increase crop yield by enhancing drought resistance and the combined application of rhizobacteria (RB) and exogenous cytokinin is more favorable than RB or CK alone [140]. In addition, the treatment of exogenous cytokinin could enhance drought tolerance by improving growth traits and yield of maize at the growing stage [148]. Similar to salt stress, we can spray exogenous cytokinins on plants to increase their drought tolerance.

## 5. Conclusions and Prospects

Cytokinins are a vital class of plant hormones and play an important role in plant response to stress. In this review, we reviewed the research progress of cytokinin in plant response to stress such as heat stress, cold stress, salt stress, and drought stress. As we know, plants have common responses (for example producing ROS) under different stresses, which make it possible that we can use the mechanism of cytokinin alleviating one stress for other stresses. Cytokinin levels in plants are closely related to plant stress tolerance. Therefore, we can increase the stress tolerance of plants by application exogenous cytokinins or reducing cytokinin in plants through genetic mutation and it is important to study their mechanisms in plant stress tolerance and increasing their application.

### 5.1. Cytokinin Response Factors (CRFs) Are Key Factors Involved in the Role of Cytokinin in Abiotic Stress

CRFs are a family of transcription factor that regulate plant growth and development and participate in plant response to abiotic stress [113]. Studies have showed that CRFs respond to cytokinin and cold, salt, drought, and oxidative stress [58,78,114,139], which showed that CRFs may integrate hormone signals with environmental conditions [113]. Therefore, it is important to reveal the function of CRFs to understand the roles of cytokinins involved in plant responses to abiotic stresses.

### 5.2. Learning from Different Stresses

Cytokinins participate in improving plant tolerance to stress. As we know, all of the abiotic stresses inhibit plant growth and cause the production of ROS, which showed that there is stress crosstalk between different stresses. As discussed in this review, cytokinin and CRFs regulate plant tolerance to stresses. Therefore, mechanisms of alleviating one stress in plants which involve cytokinin can be applied to other types of stressors, which can accelerate the process of cytokinin’s involvement in plant stress. Furthermore, hypotheses can be made based on one type of stress for another type. For example, we can hypothesize that the high expression of *CRF2* under cold stress may be an adaptive mechanism under cold stress, which may also be observed due to salt stress.

### 5.3. Enhancing Practical Application

Cytokinins have many applications in practical production, such as promoting rooting, growth, photosynthesis, and other physiological processes under stress. Compared with using molecular means to change the endogenous cytokinin, directly spraying exogenous cytokinins is a quick and effective way for plants to survive stress. Therefore, understanding the role of cytokinins in plant tolerance to stress is valuable in practical applications and we can increase the stress tolerance of plants by spraying exogenous cytokinins.
